# The implementation process of the Workload Indicators Staffing Need (WISN) method by WHO in determining midwifery staff requirements in Greek Hospitals

**DOI:** 10.18332/ejm/100559

**Published:** 2019-01-23

**Authors:** Fotini Gialama, Maria Saridi, Panagiotis Prezerakos, Yiannis Pollalis, Xenofon Contiades, Kyriakos Souliotis

**Affiliations:** 1 Department of Social and Educational Policy, University of Peloponnese, Corinth, Greece; 2 Department of Nursing, University of Peloponnese, Sparti, Greece; 3 Department of Economics, University of Piraeus, Athens, Greece; 4 Department of Public Administration, Panteion University, Athens, Greece

**Keywords:** hospital, health workforce planning, staffing, midwifery, workload indicators

## Abstract

**INTRODUCTION:**

One of the greatest challenges in the healthcare field is planning the health workforce under limited financial resources while being fully capable of responding to an affordable, fair and efficient healthcare system. This study aimed to demonstrate the implementation process of the health workforce planning tool ‘Workload Indicators of Staffing Needs’ introduced by the World Health Organization.

**METHODS:**

A descriptive and cross-sectional study was carried out at four (two public and two private) hospitals in Greece, to estimate midwifery staff requirements at ward level during 2015–2016, using the WISN software tool. Focus group discussions, structured interviews and annual service statistics from the hospitals’ records were used to obtain data.

**RESULTS:**

Results for both private hospitals showed a shortage in the number of midwives. However, after combining the interpretation of the results, as indicated by the WISN methodology and the structured interviews, current and required staffing is in balance in both. On the other hand, both public hospitals indicate a surplus of midwives (1.83 and 1.33 ratios for the General hospitals in Korinthos and Kalamata, respectively).

**CONCLUSIONS:**

This study demonstrated the implementation process of the WISN methodology through its application in midwifery staff at four hospitals in Greece and confirmed its usefulness in estimating staffing requirements. The application of the WISN methodology should be viewed as a vital tool in assessing overstaffing and understaffing through the estimation of workload pressure among different categories of health staff, thus providing the basis for effective health workforce redistribution in Greece.

## INTRODUCTION

Throughout the world countries are trying to regulate and adapt their health systems in order to ensure real and sustainable improvements in their populations’ health status as set forth in the United Nation’s Millennium Development Goals^1^. One of the most important components for the sustainability, functioning and performance of the labour-intensive health sector is the health workforce (or human resources for health) and the way it is planned and managed under limited financial resources while being fully capable of responding to an affordable, fair and efficient healthcare system^2,3^. Consequently, the process of health workforce planning that according to Hall and Mejia^4^ is ‘the process of estimating the number of persons and the kind of knowledge, skills and attitudes they need to achieve predetermined health targets and ultimately health status objectives’, is fundamental in ensuring good quality healthcare, influencing a population’s health status and ensuring the sustainability of healthcare systems across the world. Nonetheless, planning the health workforce is a challenging process. On the one hand, the changes in the sociodemographic, epidemiological cultural and social profiles of the population affect the need for health services, and on the other hand, they affect the health workforce’s composition that in turn has an impact on labour market participation and productivity^5^. These challenges not only have been deepened by the financial crisis and the subsequent fiscal austerity policies introduced in many countries across the world, but they have also increased concerns about their potential effects on public health and health systems overall^6,7^.

Given the aforementioned challenges, health workforce planning with an emphasis on the measurement of workforce needs is vital in the health sector. Traditionally, four different methods have been used for health workforce planning and for measuring workforce requirements. These are: the workforce-to-population ratio method, the health needs method, the service demands method and the service targets method^8^. Though these methods have been sufficient to tackle overall staffing requirements, they have many disadvantages that affect the demand for services in an area and at individual facilities, and ultimately, they affect the staffing levels that are actually required to meet the demand. One major disadvantage of these methods is the inability to take into consideration wide local variations that are found within every country, such as the different levels and patterns of morbidity in different locations, the ease of access between different facilities, the patient attitudes in different parts of the country to services provided and the local economic circumstances^9^.

In an attempt to address these constraints and find a suitable method for determining the optimal number and distribution of health workforce at health facilities at all levels from local to national, the World Health Organization (WHO) introduced the Workload Indicators of Staffing Need (WISN) method, developed by Shipp in 1998^9^. The WISN method is based on a health worker’s workload, with activity (time) standards applied for each workload component in order to rationally determine the number and type of staff required in a given health facility. It can be used not only in assessing and determining the required number of a particular staff category in a given health facility but also in assessing the workload pressure of each and every staff in that facility. The WISN method as a human resource management tool is able to calculate the optimal allocation and distribution of staff geographically and functionally between different types of health facilities or health services in a country as a whole, or a province, district, area, etc., according to the volume of services being delivered and the types of staff that deliver these services. In addition, the WISN method can estimate the optimal staffing patterns, categories and numbers for health facilities in accordance to local conditions such as morbidity, access to services and patient attitudes that traditional methods have failed to consider^9,10^.

Apart from the WISN method, literature revealed other alternatives that have also been used over the years for the calculation of workforce requirements. For example, Faulkner^11^ has proposed a five-step needs-based approach to estimating psychiatric workforce requirements that are supplemented by a simple formula for calculations (number of patients needing psychiatric treatment × amount of psychiatric treatment required per patient / amount of direct treatment time provided per psychiatrist = number of psychiatrists required). Dreesch et al.^[Bibr cit0001]1^ have developed an approach to estimating human resource requirements based on the time needed to address health deficits of the population^1^. Hagopian et al.^12^ have produced a demand-driven staffing model using spreadsheet technology, based on treatment protocols for HIV-positive patients to estimate personnel needs.

Compared to the above alternatives for workforce planning and staffing, experience has shown that the WISN method is easier to comprehend and use, much simpler and its information system is consistent and reliable^13^. Its simplicity lies in the software that has been developed relatively recently that can be used to facilitate WISN staffing calculations. In general, the WISN method is a complete and comprehensive human resource management tool that provides health managers with a systematic way to make staffing decisions in order to plan and manage their valuable human resources appropriately and effectively^10^.

In light of the above, this study aimed to explain and demonstrate the implementation process of the health workforce planning tool WISN, through its application in midwifery staff at four hospitals in Greece.

## METHODS

### Study design and setting

We conducted a descriptive and cross-sectional study during 2015 at two public and two private hospitals in Greece that responded positively to our request for permission. Apart from one hospital that is located in Athens. Attica, the rest three hospitals are located in the wider geographical area of the Peloponnese (Korinthos, Kalamata and Patra) and are within the research scope of the University of Peloponnese, on behalf of which the survey was conducted.

### Study population

Given the study aim of demonstrating the implementation process of the WISN method, the sample intentionally included only midwives who are the staff category that is used in the WISN users’ manual as an example for the implementation process.

### Data collection

Data were collected through focus group discussions and structured interviews and by reviewing annual service statistics from each hospital’s records. The structured interviews included a set of structured questions in order to obtain data on available staff time, workload components (i.e. Health Service Activities, Support and Additional Activities) and staff time spent on each activity (i.e. Activity Standards). Annual service statistics provided information on the annual number of deliveries (births), cesarean sections, gynaecological surgeries and newborns.

To ensure validity and reliability of results it was decided that the participants of the focus group discussion should have many years of work experience as midwives (at least ten years) and should be familiar with the Greek legislation and in particular with the Law 2539/1953 ‘Permission to practice Midwifery and Midwifery Training’ and Presidential Decree 351/1989 regarding midwives’ duties and activities.

Based on the above inclusion criteria, the focus group discussion consisted of eight participants and more specifically the four heads of the Nursing Departments and the four supervisor midwives of the Maternity Clinics from each hospital.

### WISN Procedure

Based on the WISN user’s manual the WISN method consists of seven steps in calculating staff requirements^10^:

#### Step 1

The first step is the estimation of Available Working Time (AWT), which is the time a health worker has available in one year to do his/hers work, taking into account authorized and unauthorized absences. The formula used to calculate AWT is: [A-(B+C+D+E)] × F, were A is the number of possible working days in a year; B is the number of days off for public holidays in a year; C is the number of days off for annual leave in a year; D is the number of days off due to sick leave in a year; E is the number of days off due to other leave, such as training, etc., in a year, and F is the number of working hours in one day.

#### Step 2

The second step is the definition of the workload components, which include health services, support and additional activities, i.e. work activities that take up most of a health worker’s daily working time.

#### Step 3

The third step is setting activity standards, which is the time necessary for a well-trained, skilled and motivated worker to perform an activity to professional standards in the local circumstances. Activity standards are reported in terms of rate of unit time and are divided into service standards for health service activities and allowance standards for support and additional activities.

#### Step 4

The fourth step is the establishment of standard workload, which is the amount of work within a health service workload component that one health worker can do in a year. The formula for calculating standard workload is AWT divided by unit time or multiplied by the rate of working.

#### Step 5

The fifth step is the calculation of allowance factors. The allowance standards mentioned at Step 3 can be categorized in two types: Category allowance standards (CAS) for support activities that all members of a staff category perform, and Individual Allowance Standards (IAS) for additional activities that only certain staff categories perform. The Category Allowance Factor (CAF) is a multiplier that is used to calculate the total number of health workers, required for support and health service activities. The formula used is CAF=1/[1-(Total CAS/100)]. The Individual Allowance Factor (IAF) is the staff required to cover additional activities of certain cadre members. IAF is calculated by dividing the annual total IAS by the AWT.

#### Step 6

The sixth step is the determination of the exact staff requirement by multiplying the total required staff for health service activities and CAF and then adding IAS to it.

#### Step 7

The seventh step is the analysis and interpretation of the above results.

### Data analysis and interpretation

All data collected from the interviews and annual service statistics were analyzed using the WISN software (English version 1.0.15.102). The results that are generated from the WISN software can be interpreted and analyzed using two ways, the difference and the ratio. The WISN difference compares the difference between current and required staffing levels allowing to identify understaffing or overstaffing. On the other hand, the WISN ratio divides current to required staff allowing to assess the workload pressure that staff experience in daily work in a health facility.

### Permissions and ethical approval

Ethical approval was obtained from the participating hospitals, as well as the WHO Permissions Management and Reprint Rights Office for the use of the WISN software.

## RESULTS

Following the steps in the previous section, the AWT for midwives was calculated at all four health facilities and estimated to be 1.608 hours/midwife, based on the formula [A-(B+C+D+E)] × F; were A is 260, the number of possible working days in a year; B is 12, the number of days off for public holidays in a year; C is 22, the number of days off for annual leave in a year; D is 25, the number of days off due to sick leave in a year; E is zero, the number of days off due to other leave; and F is 8, the number of working hours in one day.

Workload components for health service, support and additional activities were established and classified through the consultations with the focus groups and the structured interviews with the head of the Nursing Department and the supervisor midwife of the maternity ward of each hospital. As it is recommended by the WISN users’ manual four to five health service activities and three to four support activities are usually enough since they occupy most of the daily working time. On this note, the resulting workload components for each unit of each hospitals’ maternity clinic are demonstrated in Table 1.

**Table 1:** Defining workload components

*Units*	*Health service activities*	*Support activities*	*Additional activities*
Postnatal	Prenatal Care Admitting patients for delivery Admitting patients for caesarian Admitting patients for other gynecological surgeries		
Labor	Deliveries Postnatal follow-up Newborn care Waiting room for scheduled caesarian	Educational programmes Staff meetings Handing over shifts Educational programmes	Establishing monthly working program and allocating staff in wards planning annual leave Ordering drug and supplies Executive staffs meetings Ward rounds
Surgery	Caesarians Newborn care Recovery		
Antenatal	Receiving and admitting patients Antenatal care Newborn care		

One of the most challenging and important steps in the WISN methodology is estimating the working time that each health service activity takes if it is performed well (i.e. the service standard). Working time will allow defining standard workload, which is the amount of work within a health service workload component that one health worker can do in a year. Table 2 presents the working time and the standard workload of each health service activity in each unit at all four hospitals.

**Table 2:** Defining service standards

*Unit Activity*	*Iaso*	*Olympion*	*General Hospital of Korinthos*	*General Hospital of Kalamata*
***Postnatal Unit***				
Prenatal Care	45m/p or 1.33 p/h SW=2.144p	45m/p or 1.33 p/h SW=2.144p	40m/p or 1.5p/h SW=2412p	
Admitting patients for delivery	35m/p or 1.71 p/h SW=2756.57p	30m/p or 2 p/h SW=3216p	40m/p or 1.5p/h SW=2412p	35m/p or 1.7p/h SW=2756.57p
Admitting patients for caesarian	30m/p or 2p/h SW=3216p	20m/p or 3p/h SW=4824p	20m/p or 3p/h SW=4824p	30m/p or 2 p/h SW=3216p
Admitting patients for other gynecological surgeries	25m/p or 2.4p/h SW=3859.2p	20m/p or 3p/h SW=4824p		25m/p or 2.4p/h SW=3859.2p
***Labor Unit***				
Deliveries	8h/p SW=201p	8h/p SW=201p	8h/p SW=201p	8h/p SW=201p
Postnatal follow-up	2h/p SW=804p	2h/p SW=804p	2h/p SW=804p	4h/p SW=402p
Newborn care	3h/nb SW=536nb	3h/nb SW=536nb	2h/nb SW=804nb	2h/nb SW=804nb
Waiting room for scheduled caesarian	30m/p or 2p/h SW=3216p	20m/p or 3p/h SW=4824p		30m/p or 2p/h SW=3216p
***Surgery Unit***				
Caesarians	70m/p or 0.85p/h SW=1378.29p	70m/p or 0.85p/h SW=1378.29p	4h/p SW=402p	1h/p SW=1608p
Newborn care	3h/nb SW=536nb	3h/nb SW=536nb	2h/p SW=804nb	2h/p SW=804nb
Recovery	2h/p SW=804p	2h/p SW=804p	2h/p SW=804p	2h/p SW=804p
***Antenatal Unit***				
Receiving and admitting patients	20m/p or 3p/h SW=4824p	30m/p or 2p/h SW=3216p	30m/p or 2p/h SW=3216p	20m/p or 3p/h SW=4824p
Antenatal care	200m/p SW=483p	200m/p SW=483p	200m/p SW=483p	200m/p SW=483p
Newborn care	200m/nb SW=483nb	200m/nb SW=483nb	200m/nb SW=483nb	200m/nb SW=483nb

Service standards are expressed either as unit time or rate of working. p=patients, m=minutes, h=hour, SW= standard workload, nb=newborn.

Similarly, working time for support and additional activities, (i.e. category allowance standards and individual allowance standards, respectively), was recorded as demonstrated in Table 3.

**Table 3:** Category allowance standards and individual allowance standards

*Workload Components*	*Working time*							
	*Iaso*		*Olympion*		*General Hospital of Korinthos*		*General Hospital of Kalamata*	
***Support Activities***								
Educational programmes	4 h/m		-		4 h/m		1 h/m	
Staff meetings	2 h/m		1 h/m		1 h/m		-	
Handing over shifts	1 h/d		1 h/d		1 h/d		1 h/d	
***Additional Activities***								
Establishing monthly working program and allocating staff in wards	2 workers	6 h/m	1	6 h/m	1 worker	1 h/w	1 worker	1 h/w
Planning annual leaves	1 worker	16 h/y	1	16 h/y	1 worker	5 h/y	1 worker	5 h/y
Ordering drug and supplies	1 worker	2 h/w	1	2 h/w	1 worker	2 h/w	1 worker	2 h/w
Executive staff meetings	1 worker	2 h/m	1	2 h/m	1 worker	1 h/d	1 worker	1 h/m
Ward rounds	1 worker	1 h/d	1	1 h/d	1 worker	1 h/w	1 worker	1 h/d

h/d=hour/day; h/m=hour/month; h/y=hour/year; h/w= hour/week

In order to determine the required midwifery staff for each health facility, the annual service statistics from the previous year were used (i.e. annual workload). Finally, all the data collected were transferred to the WISN software (English version 1.0.15.102). The number of midwives required for each hospital, respectively, is demonstrated in Tables 4–7, which summarizes all the steps as proposed by the WSIN method. The required number of staff, as shown in Tables 4–7, can result by multiplying the total required staff of health service activities of each ward with the CAF and then by adding the IAF. Both factors are calculated automatically by the software when entering all data needed.

**Table 4:** Determining midwifery requirements, based on WISN

*IASO Hospital (AWT= 1.608 hours/year)*			
*Health service activities of all cadre members*	Annual Workload*	*Standard Workload*	*Required number of staff members*
***Postnatal Unit***			
Prenatal Care	900	2144 patients	0.42
Admitting patients for delivery	816	3216 patients	0.25
Admitting patients for caesarian	589	4824 patients	0.12
Admitting patients for other gynecological surgeries	32	4824 patients	0.01
Α1. Total required staff for health service activities for Postnatal Unit			**0.8**
***Surgery Unit***			
Caesarians	589	1378.29 patients	0.43
Newborn care	608	536 newborns	0.13
***Surgery Unit***			
Recovery	589	804 patients	0.73
Α2. Total required staff for health service activities for Surgery Unit			**2.29**
***Antenatal Unit***			
Receiving and admitting patients	816	3216 patients	0.25
Antenatal care	816	482.4 patients	1.69
Newborn care	1443	482.4 newborns	2.99
Α3. Total required staff for health service activities for Antenatal Unit			**4.93**
***Labor Unit***			
Deliveries	227	201 patients	1.13
Postnatal follow-up	227	804 patients	0.28
Newborn care	835	536 newborns	1.56
Waiting room for scheduled caesarian	589	4824 patients	0.12
Α4. Total required staff for health service activities for Labor Unit			**3.09**
***Support activities of all cadre members***	***CAS (Actual working time)***	***CAS (Percentage working time)***	***-***
Staff Meetings	1 hour/month	0.75%	-
Handing over shifts	1 hour/day	12.5%	-
**Total CAS percentage**		**13.25%**	-
**Β. Category allowance factor: 1/[1-(total CAS percentage /100)]**		**≈1.16**	-
***Additional activities of certain cadre members***	***Number of staff members performing the work***	***IAS (Actual working time per person)***	***Annual IAS (for all staff performing activity)***
Establishing monthly working program and allocating staff in wards	1	6 hours/month	72 hours
Planning annual leaves	1	16 hours/year	16 hours
Ordering drug and supplies	1	2 hours/week	104 hours
Executive staff meetings	1	2 hours/month	24 hours
Ward rounds	1	1 hour/day	201 hours
**Total IAS in a year**			**417 hours**
**C. Individual allowance factor (Annual total IAS/AWT)**			**≈0.34**
**Total required number of staff, based on WISN (A × B + C)**			**1.17** (Postnatal Unit) **2.9** (Surgery Unit) **5.97** (Antenatal Unit) **3.83** (Labor Unit)

* Data from Annual Statistics

**Table 5:** Determining midwifery requirements, based on WISN

*OLYMPION Hospital (AWT= 1.608 hours/year)*			
*Health service activities of all cadre members*	Annual Workload*	*Standard Workload*	*Required number of staff members*
***Postnatal Unit***			
Prenatal Care	900	2144 patients	0.42
Admitting patients for delivery	816	3216 patients	0.25
Admitting patients for caesarian	589	4824 patients	0.12
Admitting patients for other	32	4824 patients	**0.01**
Α1. Total required staff for health service activities for Postnatal Unit			**0.8**
***Surgery Unit***			
Caesarians	589	1378.29 patients	0.43
Newborn care	608	536 newborns	0.13
Recovery	589	804 patients	0.73
Α2. Total required staff for health service activities for Surgery Unit			**2.29**
***Antenatal Unit***			
Receiving and admitting patients	816	3216 patients	0.25
Antenatal care	816	482.4 patients	1.69
Newborn care	1443	482.4 newborns	2.99
Α3. Total required staff for health service activities for Antenatal Unit			**4.93**
***Labor Unit***			
Deliveries	227	201 patients	1.13
Postnatal follow-up	227	804 patients	0.28
Newborn care	835	536 newborns	1.56
Waiting room for scheduled caesarian	589	4824 patients	0.12
Α4. Total required staff for health service activities for Labor Unit			**3.09**
***Support activities of all cadre members***	***CAS (Actual working time)***	***CAS (Percentage working time)***	***-***
Staff Meetings	1 hour/month	0.75%	-
Handing over shifts	1 hour/day	12.5%	-
**Total CAS percentage**		**13.25%**	-
**Β. Category allowance factor: 1/[1-(total CAS percentage /100)]**		**≈1.16**	-
***Additional activities of certain cadre members***	***Number of staff members performing the work***	***IAS (Actual working time per person)***	***Annual IAS (for all staff performing activity)***
Establishing monthly working program and allocating staff in wards	1	6 hours/month	72 hours
Planning annual leaves	1	16 hours/year	16 hours
Ordering drug and supplies	1	2 hours/week	104 hours
Executive staff meetings	1	2 hours/month	24 hours
Ward rounds	1	1 hour/day	201 hours
**Total IAS in a year**			**417 hours**
**C. Individual allowance factor (Annual total IAS/AWT)**			**≈0.34**
**Total required number of staff, based on WISN (A × B + C)**			**1.17** (Postnatal Unit) **2.9** (Surgery Unit) **>5.97** (Antenatal Unit) **3.83** (Labor Unit)

* Data from Annual Statistics

**Table 6:** Determining midwifery requirements, based on WISN

*General Hospital of Korinthos (AWT= 1.608 hours/year)*			
*Health service activities of all cadre members*	Annual Workload*	*Standard Workload*	*Required number of staff members*
***Postnatal Unit***			
Prenatal Care	258	2412 patients	0.11
Admitting patients for delivery	136	2412 patients	0.06
Admitting patients for caesarian	122	4824 patients	0.03
Α1. Total required staff for health service activities for Postnatal Unit			**0.20**
***Surgery Unit***			
Caesarians	122	402 patients	0.30
Newborn care	122	804 newborns	0.15
Recovery	122	804 patients	0.15
Α2. Total required staff for health service activities for Surgery Unit			**0.60**
***Antenatal Unit***			
Receiving and admitting patients	258	3216 patients	0.08
Antenatal care	258	482.4 patients	0.53
Newborn care	258	482.4 newborns	0.53
Α3. Total required staff for health service activities for Antenatal Unit			**1.14**
***Labor Unit***			
Deliveries	136	201 patients	0.68
Postnatal follow-up	136	804 patients	0.17
Newborn care	136	804 newborns	0.17
Waiting room for scheduled caesarian	589	4824 patients	0.12
Α4. Total required staff for health service activities for Labor Unit			**1.02**
***Support activities of all cadre members***	***CAS (Actual working time)***	***CAS (Percentage working time)***	***-***
Educational programmes	4 hours/month	2.98%	-
Staff Meetings	1 hour/month	0.75%	-
Handing over shifts	1 hour/day	12.5%	-
**Total CAS percentage**		**16.23%**	-
**Β. Category allowance factor: 1/[1-(total CAS percentage /100)]**		**≈1.2**	-
***Additional activities of certain cadre members***	***Number of staff members performing the work***	***IAS (Actual working time per person)***	***Annual IAS (for all staff performing activity)***
Establishing monthly working program and allocating staff in wards	1	1 hour/month	52 hours/year
Planning annual leaves	1	5 hours/year	5 hours/year
Ordering drug and supplies	1	2 hours/week	104 hours/year
Ward rounds	1	1 hour/day	201 hours/year
**Total IAS in a year**			**362 hours**
**C. Individual allowance factor (Annual total IAS/AWT)**			**≈0.21**
**Total required number of staff, based on WISN (A × B + C)**			**0.45** (Postnatal Unit) **0.93** (Surgery Unit) **1.58** (Antenatal Unit) **1.44** (Labor Unit)

* Data from Annual Statistics

**Table 7:** Determining midwifery requirements, based on WISN

*General Hospital of Kalamata (AWT= 1.608 hours/year)*			
*Health service activities of all cadre members*	Annual Workload*	*Standard Workload*	*Required number of staff members*
***Postnatal Unit***			
Prenatal Care	214	2756.57 patients	0.08
Admitting patients for delivery	361	3216 patients	0.11
Admitting patients for caesarian	725	3859.2 patients	0.19
Α1. Total required staff for health service activities for Postnatal Unit			**0.38**
***Surgery Unit***			
Caesarians	361	1608 patients	0.22
Newborn care	361	804 newborns	0.45
Recovery	361	804 patients	0.45
Α2. Total required staff for health service activities for Surgery Unit			**1.12**
***Antenatal Unit***			
Receiving and admitting patients	575	482.4 patients	0.12
Antenatal care	575	482.4 patients	1.19
Newborn care	575	482.4 newborns	1.19
Α3. Total required staff for health service activities for Antenatal Unit			**2.50**
***Labor Unit***			
Deliveries	214	201 patients	1.06
Postnatal follow-up	214	402 patients	0.53
Newborn care	214	804 newborns	0.27
Waiting room for scheduled caesarian	361	3216 patients	0.11
Α4. Total required staff for health service activities for Labor Unit			**1.97**
***Support activities of all cadre members***	***CAS (Actual working time)***	***CAS (Percentage working time)***	***-***
Educational programmes	1 hour/month	0.75%	-
Handing over shifts	1 hour/day	12.5%	-
**Total CAS percentage**		**13.25%**	-
**Β. Category allowance factor: 1/[1-(total CAS percentage /100)]**		**≈1.1**	-
***Additional activities of certain cadre members***	***Number of staff members performing the work***	***IAS (Actual working time per person)***	***Annual IAS (for all staff performing activity)***
Establishing monthly working program and allocating staff in wards	1	1 hour/week	52 hours/year
Planning annual leaves	1	5 hours/year	5 hours/year
Ordering drug and supplies	1	2 hours/week	104 hours/year
Executive staff meetings	1	1 hour/month	12 hours
Ward rounds	1	1 hour/week	201 hours/year
**Total IAS in a year**			**374 hours**
**C. Individual allowance factor (Annual total IAS/AWT)**			**≈0.23**
**Total required number of staff, based on WISN (A × B + C)**			**0.64** (Postnatal Unit) **1.52** (Surgery Unit) **3.13** (Antenatal Unit) **2.51** (Labor Unit)

* Data from Annual Statistics

In order to estimate the total number of midwives for each hospital, the number of the required staff of each ward (as shown in Tables 4–7) at each hospital was summed up. The final aggregated results of the required staff along with the workload indicators per hospital are shown in Table 8.

**Table 8:** WISN aggregated results

*Health Facility*	*Existing staff*	*Required staff*	*Difference in staff (existing-required)*	*Workforce problem*	*WISN Ratio*	*Workload Pressure*
Iaso Hospital	97	117	-19	Shortage	0.83	High
Olympion Hospital	14	15	-1	Shortage	0.93	Moderate
General Hospital of Korinthos	11	6	5	Surplus	1.83	None
General Hospital of Kalamata	12	9	3	Surplus	1.33	None

More specifically, the results show the calculated required staff for optimum staffing, the differences between the actual and the calculated required staffing (shortage or surplus) and the WISN ratio. The WISN ratio shows the workload pressure, in other words, the amount of pressure each midwife is undergoing to cope with the annual workload (i.e. annual statistics) and reveals the under or overstaffing in each specific hospital. According to the WISN method, if the WISN ratio is 1.00, then the calculated staff is in balance, meaning that is just sufficient to meet the workload of that particular health facility. On the other hand, if the WISN ratio is <1.00, the staff is under workload pressure, and if the ratio is >1.00, the staff is more than sufficient to cope with the workload.

## DISCUSSION

Health workforce has a central and significant role in delivering quality healthcare services to the population. Health policy makers are responsible in managing effectively and efficiently the health workforce in order to ensure that the right number of healthcare workers, with the right knowledge, skills, attitudes and qualifications are performing the correct tasks in the right place at the appropriate time in order to achieve predetermined health targets^14,15^. Nonetheless, health workforce planning should be an integrated rather than a solely technical process incorporating all demographic, epidemiological, cultural and social forces that affect both health service provision and health workforce demand^1,2^.

In search of a suitable method for health workforce planning and specifically for determining the required number of health workers, WHO introduced the WISN method. Compared to the traditional methods of staffing using population-to-staff facility-based ratios, the WISN method is simple and comprehensible to use; it can be applied nationally, regionally or to a single health facility and to any type of health worker and it is realistic, providing practical targets for budgeting and resource allocation^9,10^.

The present study was conducted in order to demonstrate the implementation process of the WISN method. In doing so, the WISN software was used to estimate the optimal midwifery staff requirements at four hospitals (two public and two private) in Greece. Results from the application of the WISN method can be categorized between public and private hospitals. For private hospitals (IASO and Olympion), output from the use of the WISN method indicates a shortage in the number of midwives and a WISN ratio below 1.00 (0.83 for IASO Hospital and 0.93 for Olympion Hospital). However, after combining the interpretation of the results as indicated by the WISN methodology and the structured interviews, in both private hospitals, current and required staffing is in balance. On the other hand, both public hospitals indicate a surplus of midwives with WISN ratios of 1.83 and 1.33 for the General hospital in Korinthos and Kalamata, respectively. The results from public hospitals reveal the inadequate planning and staffing of the health workforce in the Greek public health sector, which is based on the number of beds of each hospital rather than its output^16^.

At present, there is no other study calculating staffing requirements based on workload in Greece, despite the fact that there are numerous of studies examining the impact of workload pressure among health staff, especially nursing, both in its working conditions and in the provided services^17,18^. On the contrary, there is a substantial number of studies concerning the WISN implementation and adoption, though mainly in developing countries. More specifically, the WISN method has been used in Papua New Guinea, Kenya, the United Republic of Tanzania, Sri Lanka, Bahrain, Egypt, Hong Kong, Oman, Sudan, Turkey, Uganda, Indonesia and Mozambique, Iran, Abu Dhabi, among others^13,19-39^.

Although the direct comparison of our findings with those from international literature is not possible due to the heterogeneity of the staff category, sample size, setting, (hospitals vs health centers), etc., nonetheless, all studies have succeeded in highlighting the importance of the implementation of such a tool as well as its usefulness in policy-making decisions regarding recruitment, distribution, training and education, and job burnout issues. In addition, the above case studies from countries that differ in terms of economic development and health systems, indicated the WISN methods’ flexibility and ability to adapt to each country’s individual conditions and circumstances and also the ability to use WISN not only for health workforce planning and staffing in health facilities that already exist and are in operation, but also in the establishment of new departments or clinics within health facilities^39^.

Though the WISN method has been extensively used and implemented in various countries throughout the world as stated above, this is the first attempt at establishing the optimal staffing level of any cadre (i.e. staff category) in Greece, employing widely used international methodologies. Yet, there are several studies concerning the importance of health workforce planning and the need of health workforce sustainability in Greece, especially in regard to the relatively recent Greek economic crisis that has had a major impact on the healthcare system as a whole, revealing a complete absence of planning and the need for healthcare workforce management tools^7,40-42^.

As part of the reforms that Greece had to undergo due to the austerity measures imposed by the economic crisis, the Greek government is making important efforts towards the development of a health workforce strategy and is committed to investing resources towards the goal of universal health coverage with an appropriately skilled, adequate, supported and deployed health workforce. The long-term goal is to improve the population’s health and access to health services, the effectiveness of the health system and the quality of care.

Based on the above, there is an apparent need in developing and using evidence-based staffing norms (staffing-standards) using the WHO WISN method, towards the realization of a health workforce strategy, as has been done already by other countries that are also undergoing health sector reforms^43^.

In that sense, this study can be viewed as a pilot study for the implementation of the WISN method at a national level. More specifically, the Greek National Health System can benefit from the use of the WISN method not only in estimating the optimum staff number, but also in precisely defining the workload components (i.e. work activities) that take up most of a health worker’s daily working time and also in allocating the appropriate time to service provision. In doing so, new streamlined protocols can be created for the tasks and the time needed of individual health services performed not only for midwives but also for other staff categories in health facilities (i.e. hospitals, health centres, etc.), in Greece.

Limitations of this study include that it was conducted using statistical data retrospectively gathered from the preceding year; thus, the accuracy of this study’s results is directly linked to the accuracy of the service statistics of each hospital. If record keeping is not well maintained the resulting WISN metrics may not accurately reflect the required staffing levels and the workload of each staff.

## CONCLUSIONS

This study demonstrated the implementation process of the WISN methodology, using as an example the estimation of midwifery staff in the maternity wards of four hospitals in Greece. Through this application, we sought to confirm the usefulness of the WISN methodology as a health workforce planning tool in estimating staffing requirements in hospitals. More specifically, we estimated both the required number of midwives in order to cope with the workload of each hospital and the workload pressure of each midwife in these hospitals. Results from our study can be used in order to assess overstaffing and/or understaffing as well as to determine workload pressure among midwives and other staff categories in various healthcare settings, hence providing a basis for effective health workforce reallocation without compromising the quality of health services. Overall the adoption and application of the WISN methodology should be viewed as a vital tool in improving health workforce planning and management in healthcare settings, by facilitating adequate staffing and appropriately utilizing staff categories according to their professional scope of practice and actual daily workload.
